# Effects of particulate matter on atherosclerosis: a link via high-density lipoprotein (HDL) functionality?

**DOI:** 10.1186/s12989-020-00367-x

**Published:** 2020-08-04

**Authors:** Siri A. N. Holme, Torben Sigsgaard, Jørn A. Holme, Gitte Juel Holst

**Affiliations:** 1 Department of Clinical Medicine, Faculty of Health, Aarhus University, Aarhus, Denmark; 2 Research Unit of Environment, Occupation and Health, Department of Public Health, Aarhus University, Aarhus, Denmark; 3 Department of Environmental Health, Division of Infection Control and Environmental Health, Norwegian Institute of Public Health, Oslo, Norway

**Keywords:** Air pollution, Particulate matter, Cardiovascular disease, Atherosclerosis, Lipoproteins, High-density-lipoprotein, Oxidative stress, Inflammation

## Abstract

**Background:**

Exposure to air pollution has been associated with adverse effects on human health, and ultimately increased morbidity and mortality. This is predominantly due to hazardous effects on the cardiovascular system. Exposure to particulate matter (PM) is considered to be responsible for the most severe effects.

**Main body:**

Here we summarize current knowledge from existing epidemiological, clinical and animal studies on the influence of PM exposure on high-density lipoprotein (HDL) functionality and the potential initiation and progression of atherosclerosis. We highlight experimental studies that bring support to the causality and point to possible mechanistic links. Recent studies indicate that the functional properties of HDL are more important than the levels per se. Fine (PM_2.5–0.1_) and ultrafine (UFP) PM are composed of chemicals as well as biological elements that are redox-active and may trigger pro-inflammatory responses. Experimental studies indicate that these properties and responses may promote HDL dysfunction via oxidative pathways. By affecting protein and lipid components of the HDL particle, its anti-atherosclerotic characteristics including cholesterol efflux capacity, as well as other anti-oxidative and anti-inflammatory features might be impaired.

**Conclusion:**

Current literature suggests that PM promotes HDL dysfunction via oxidative pathways. However, as relatively few studies so far have evaluated the impact of particulate air pollution on HDL functionality, more human epidemiological as well as experimental studies are needed to strengthen any possible causal relationship and determine any relevance to atherosclerosis.

**Graphical abstract:**

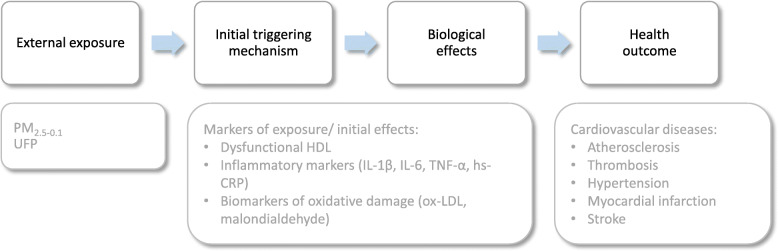

## Background

Ambient air pollution is a major public health issue, and is associated with both increased morbidity and mortality [1, 2]. Several studies confirm the link between exposure to particulate matter (PM) and the progression of cardiovascular disease (CVD) [1].

PM is a mixture of particles and liquid droplets suspended in the air, which possesses a variety of organic and inorganic substances [3]. They are classified according to their aerodynamic equivalent diameter into coarse PM_10–2.5_ (10 μm, > 2.5 μm), fine PM_2.5–0.1_ (2.5 μm, > 0.1 μm) and ultrafine particles (UFP), (< 0.1 μm). The biological effects of PM are dependent on the composition and size of the particles [3]. Exposure to PM has been linked to several biological processes being central for CVD [1], including atherosclerosis, vasomotor dysfunction, increased blood clot formation, and alterations in heart rhythm [4].

### PM-induced ROS and inflammatory reactions linked to atherosclerosis

The prevailing theory regarding the biological mechanism linking PM and CVD is an activation of inflammatory pathways and oxidative stress [2], which may contribute to an initiation of atherogenesis and promotion of atherosclerosis. Human studies show a correlation between PM exposure and increased systemic oxidative stress, based on detection of biomarkers on oxidative alterations in proteins, lipids and DNA in urine or blood [5]. The presence of oxidation in the vascular system is of great relevance, as it may imply involvement of oxidative modifications of plasma lipoproteins which are key players in atherogenesis [6]. It has been demonstrated that ambient PM exhibit pro-inflammatory effects in endothelial cells, macrophages and epithelial cells through generation of reactive oxygen species (ROS) and oxidative stress [7–9]. Furthermore, exposure to PM has been associated with increasing levels of pro-inflammatory markers including interleukin (IL)-1β, IL-6 and tumour necrosis factor (TNF)-α [1, 10].

PM has been suggested to induce atherosclerosis either by: i) activation of a lung autonomic reflex, ii) triggering inflammation in the lungs resulting in systemic “spill over” of pro-inflammatory mediators, and/or iii) the translocation of particles or adhered constituents into circulation, thereby reacting with endothelial cells as well as components in the blood, including, lipoproteins [1, 11]. The particles’ reactivity, including surface charge and adhered chemical groups such as polycyclic aromatic hydrocarbons (PAHs) and redox-active metals, are central for these initial reaction steps, and they will thus determine the particle toxicity [11]. Recent studies propose that the key triggering events involved in PM-mediated activation of pro-inflammatory responses occur via direct interaction with molecular targets and lipid layer of cell membranes, activation of cellular receptors, and via reactive metabolites, including ROS with subsequent oxidative stress [12, 13]. Reactive electrophilic metabolites as well as ROS generated directly by PM components or via activation of specific enzymes like NADPH oxidase and myeloperoxidase (MPO) may trigger the release of pro-inflammatory cytokines. PM may also trigger macrophages to release ROS in addition to cytokines [12, 14, 15]. This may be a beneficial physiological response targeting pathogens, including bacteria, mold and virus [2]. However, if sustained over longer periods such pro-inflammatory reactions may promote detrimental effects on the vascular wall.

### Atherosclerosis and the role of lipoproteins

**Fig. 1:**
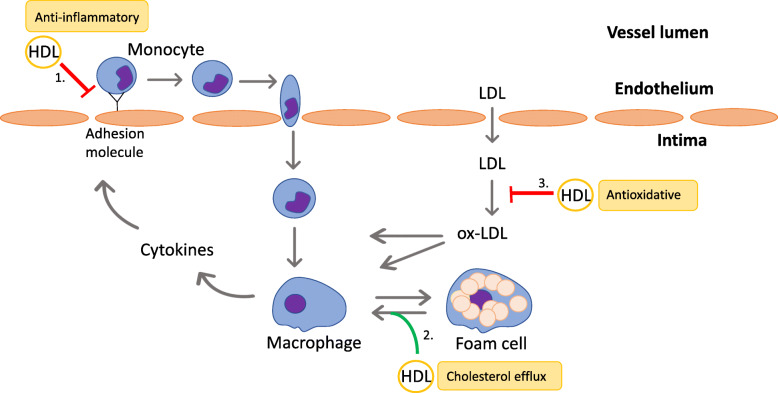
Anti-atherogenic features of HDL. Atherosclerosis is an inflammatory condition initiated by accumulation and subsequent oxidation of LDL in the arterial intima. Ox-LDL promotes differentiation of monocytes into macrophages that scavenge ox-LDL and transform into foam cells. Macrophages express cytokines which stimulate the endothelium to express adhesion molecules leading to interaction with circulating monocytes. 1) HDL inhibits expression of adhesion molecules on the epithelium and thereby inhibits monocyte chemotaxis and formation of foam cells. 2) HDL mediates cholesterol efflux and thereby decreases the accumulation of foam cells. 3) The primarily antioxidative effect of HDL is inhibition of oxidation of LDL. HDL: high-density lipoprotein; LDL: low-density lipoprotein; ox-LDL: oxidized low-density lipoprotein. (*Inspired by Barter* et al. *2004)*

Atherosclerosis is well-known as a chronic, low-grade inflammatory process in the arterial wall that predisposes to acute CVDs like myocardial infarction and stroke [16]. Central steps in atherogenesis include development of: i) endothelial dysfunction, ii) increased expression of adhesion molecules and increased permeability, iii) deposition and oxidation of low-density lipoprotein (LDL) in the arterial intima and iv) recruitment of monocytes transforming into macrophages which scavenge the oxidized LDL (ox-LDL) and ultimately transform into foam cells. This may lead to fatty streak formation – a characteristic feature of atherosclerosis [17] (Fig. 1).

Atherosclerosis involves lipid transport characterized by excessive cholesterol in the arterial intima, a process in which the plasma lipoproteins LDL and high-density lipoprotein (HDL) play an essential role. HDL-cholesterol (HDL-C) has often been correlated with a reduced risk of CVD, whereas elevated levels of LDL-cholesterol (LDL-C) have been associated with an increased risk [18, 19].

HDL is a highly heterogenous group, consisting of components differing in size, density and composition [20], acting via numerous pathophysiological mechanisms [21]. Of great importance is the capability of mediating reverse cholesterol transport (RCT) by which HDL scavenges cholesterol from the peripheral vasculature and transports it to the liver. The cholesterol efflux capacity (CEC) is a key feature as it serves as the first step of RCT in the arterial wall. Additionally, HDL possesses significant anti-inflammatory and antioxidant activity facilitated by various enzymes (e.g. paraoxonase). The anti-inflammatory characteristics result in an inhibition of monocyte chemotaxis, while the antioxidant activity mainly provides a protective effect against ox-LDL and thereby reduces cellular uptake by the monocyte-macrophage system [22]. It has also been demonstrated that HDL has beneficial effects on platelet- and endothelial function via nitrogen oxide (NO), a potent vasodilator and anti-platelet agent [23]. Thus, HDL is evidently acting in an atheroprotective matter in multiple ways.

The oxidation of LDL plays a central role in the initiation and progression of atherosclerosis. Apart from leading to the formation of foam cells, the presence of ox-LDL also stimulates cellular proliferation, migration, necrosis and inflammatory changes [24]. The biological effects of ox-LDL have been investigated in numerous studies, while the role of oxidized HDL (ox-HDL) in the context of atherosclerosis and CVD is less known. The relationship between HDL and CVD is complex as the HDL-C levels do not necessarily depict HDL function and thereby its impact on CV health [25]. Thus, newer research has set focus on the functional aspect of the lipoprotein rather than the cholesterol component itself [26].

In recent years, the literature has been growing regarding the role of HDL functionality as a possible mechanistic explanation linking the effects of PM to atherosclerosis. However, no scientific overview of the existing knowledge has been undertaken. Therefore, we will review the current epidemiological and experimental literature on PM and HDL functionality, and summarize the present understanding of the potential mechanisms involved.

## Methods

### Search strategy and study selection

The objective was to determine the association between PM and HDL functionality by summarizing the findings of epidemiological and experimental studies. Thus, we conducted a PubMed search prior to April 3rd 2020 using the following search terms: (“Lipoproteins, HDL”[Mesh] OR “Cholesterol, HDL”[Mesh]) AND (“Particulate Matter”[Mesh] OR “Air Pollution”[Mesh] OR “Air Pollutants”[Mesh] OR “Inhalation Exposure”[Mesh]). Using this approach 67 publications were found. These were screened at the abstract level. All relevant epidemiological and human exposure studies as well as animal studies assessing the effects of particulate air pollution on HDL functionality were included, resulting in a total of two epidemiological [27, 28], two clinical [29, 30] and three animal studies [31–33]. Additional search strategies were also applied focusing on keywords (combinations of keywords): (“HDL” OR “high-density lipoprotein”) AND (“PM” OR “particulate matter” OR “air pollution” OR “inhalation exposure”). This resulted in 225 publications in PubMed. By screening article abstracts, one additional animal study assessing PM and HDL functionality was found [34]. Criteria of inclusion included: general population, language in English, exposure to PM, and outcomes related to HDL functionality parameters such as cholesterol efflux capacity, anti-oxidative and anti-inflammatory properties.

## Results

In Tables 1, 2 and 3 an overview of the current studies on PM exposure and measures of various HDL functional properties is presented. Only a few studies have evaluated the impact of PM on HDL functionality. At present, there are two epidemiological studies, two clinical trials and four animal studies.

**Table 1:** Epidemiological studies on PM exposure and HDL functionality

Author, year and country	Design	Study population	Air pollutant	Exposure	Outcome	Results	Main findings
**Mathew et al. 2018, United States** [27]	Prospective follow-up study	Country: United States Age: 18–50 y (32.1 ± 9.6 y) Non-smoking adults without a history of CV disease or risk factors *n* = 50 (34 female subjects)	PM_2.5_	Personal PM_2.5_ exposure for 24 h [12,2 ± 16,9 μg/m^3^] and ambient PM_2.5_ exposure for 7 days [9,1 ± 1,8 μg/m^3^].	HDL-C level Serum cholesterol efflux capacity (CEC) HDL oxidation markers	Higher ambient PM_2.5_ exposures (per 10 mg/m^3^) were associated with reductions in CEC. Exposures were not associated with MPO-induced oxidation or other HDL-oxidation markers. Previous 24-h personal-level PM_2.5_ exposure did not impact outcomes.	Even low levels of PM_2.5_ exposure is linked to impaired HDL functionality (CEC).
**Li et al. 2019, China** [28]	Prospective follow-up study	Country: China Age: 18–50 y (23.3 ± 5.4 y) Non-smoking adults without pre-existing CV disease or risk factors n = 73 (48 female subjects)	PM_2.5_ PNC_5–50_ PNC_50–100_ PNC_100–560_ BC	Average daily concentration of PM_2.5_were 62,9 μg/m^3^ (8,1–331,0 μg/m^3^), followed up with 4 study visits during a 14-month period.	HDL-C and apoA-I levels HDL cholesterol efflux capacity (CEC) HDL antioxidative activity measured as HDL oxidation index (HOI) Metrics of systemic inflammation and oxidative stress: ox-LDL, malondialdehyde (MDA), high sensitivity C-reactive protein (hs-CRP)	Significant decreases in CEC were associated with interquartile range increases in moving average concentrations of PM_2.5_ during the 1 to 7 days before each participant’s clinic visit. Higher ambient air pollutant levels were also associated with significant reductions in circulating HDL-C and apoA-I, as well as elevations in HOI, oxidized LDL, MDA, and hs-CRP.	Higher ambient air pollution exposure was associated with impairments in HDL functionality (CEC, HOI) and parameters of oxidative stress and inflammation.

*Studies are shown in chronological order based on the year of publication*

**Table 2:** Clinical trials on PM exposure and HDL functionality

Author, year and country	Design	Study population	Air pollutant	Exposure	Outcome	Results	Main findings
**Maiseyeu et al. 2014, United States** [29]	Randomized double-blinded crossover study	Country: United States Age: 18–50 y (25.9 ± 6.6 y) Non-smoking adults without established CV disease or traditional CV risk factors *n* = 32 (16 female subjects)	PM_10–2.5_	Coarse concentrated ambient particles (CAP) [76,2 ± 51,5 μg/m^3^] in a rural location and filtered air (FA) for 2 h.	HDL mediated cholesterol efflux capacity (CEC) HDL antioxidant capacity (measured as HDL oxidation index (HOI)) Paraoxonase (PON) activity	There were no significant differences detected in CEC metrics to HDL from subjects exposed 2 h or 20 h following CAP versus FA exposures. HOI and PON activity did not differ 20-h post-CAP versus FA, respectively.	Brief inhalation of high levels of coarse PM did not acutely impair several facets of HDL functionality (CEC, HOI, PON activity).
**Ramanathan et al. 2016, United States** [30]	Randomized blinded crossover study	Country: Canada Age: 18–50 y (28 ± 9 y) Non-smoking adults without any risk for CV disease *n* = 30 (17 female subjects)	PM_2.5_	PM_2.5_ targeted at 150 μg/m^3^for 2 h on 4 different occasions at least two weeks apart.	HDL antioxidant/ anti-inflammatory capacity measured as HDL oxidation index (HOI)) Paraoxonase (PON) activity	There was a trend towards bigger ΔHOI between PM_2.5_ and FA 1 h after exposures (*p* = 0.18) but not 20 h after. This trend became significant (*p* < 0.05) when baseline HOI was lower (< 1.5 or < 2.0), indicating decreased HDL antioxidant/anti-inflammatory capacity shortly after the exposures. No significant differences in the enzymatic activity of PON-1 was observed.	Exposure to concentrated PM_2.5_ induced swift effects on HDL anti-oxidative/anti-inflammatory capacity. Changes were, however, transient and of short duration.

*Studies are shown in chronological order based on the year of publication*

**Table 3:** Experimental studies in animals on PM exposure and HDL functionality

Author, year and country	Animal models	Number of included animals	Air pollutant	Exposure including route of exposure and level	Outcome	Results	Main findings
**Araujo et al. 2008, United States** [31]	Male ApoE −/− mice (6 weeks) fed normal chow	Animals were randomly assigned to 3 groups: FA, PM_2.5_, UFP (*n* = 17/group) Controls exposed to FA: nE= 17 All: *n* = 51	UFP (< 0,18 μm) or PM_2.5_ (< 2,5 μm)	UFP (112,61 μg/m^3^) or PM_2.5_ (438,29 μg/m^3^) or FA 5 h/day, 3-days/week, 75 h (40 days). Whole-body exposure chambers in a mobile laboratory located in downtown Los Angeles.	Atherosclerotic lesions HDL-C levels HDL anti-inflammatory properties Parameters of systemic inflammation and oxidative stress (malondialdehyde (MDA), lipid peroxidation, Nrf2-induced phase II enzyme expression, antioxidant phase II enzymes (catalase, glutathione S-transferase Ya and NAD(P)H-quinone oxidoreductase 1))	Mice exposed to UFPs alone exhibited greater and more advanced lesions compared with FA- or PM_2.5_-exposed animals. Exposure to PM_2.5_ and UFP was associated with a decreased anti-inflammatory capacity of HDL (UFP greater than PM_2.5_), as well as increased hepatic MDA levels and Nrf2-regulated antioxidant genes.	Exposure to PM_2.5_ and UFP exhibited the development of dysfunctional HDL (decreased anti-inflammatory capacity) without affecting HDL-C levels.
**Li et al. 2013, United States** [32]	Male LDLR −/− mice on a high-fat diet	Controls exposed to FA: *n* = 5 All: *n* = 15	UFP (< 0,10–0,20 μm)	UFP (360 μg/m^3^) or FA for 5 h/day, 3-days/week for 10 weeks. The collection of UFPs was conducted in urban regions of Los Angeles, and animals were exposed in whole-body chambers.	Atherosclerotic lesions HDL-C level HDL antioxidative capacity (HDL oxidation index (HOI)) Paraoxonase (PON) activity Parameters of systemic inflammation and oxidative stress (hydroxyeicosatetraenoic acids (HETEs) and hydroxyoctadecadienoic acids (HODEs) serum amyloid A (SAA), tumor necrosis factor (TNF-α))	UFP exposure was associated with a greater atherosclerotic lesion size compared with FA-exposed animals. Mice exposed to UFPs developed a reduced plasma HDL-C level, PON activity, and HDL antioxidant capacity; but increased LDL oxidation, free oxidized fatty acids, triglycerides, SAA and TNF-α, accompanied by an increase in atherosclerotic lesion size.	Exposure to UFP was associated with reduced HDL antioxidant capacity, PON activity as well as HDL-C levels.
**Yin et al. 2013, United States** [33]	Male ApoE −/− mice (9 weeks)	Mice were assigned to 3 groups: DE, FA, DE + FA (*n* = 12–13/group) Controls exposed to FA: *n* = 13 All: *n* = 38	Diesel exhaust (DE) of PM_2.5_	DE at ≈250 μg/m^3^of PM_2.5_ or FA for 2 weeks. DE was generated in the exposure facility and animals were exposed in whole-body chambers.	HDL-C HDL anti-inflammatory capacity HDL antioxidative capacity Paraoxonase (PON) activity Myeloperoxidase (MPO) Malondialdehyde (MDA) Hydroxyeicosatetraenoic acids (HETEs) and hydroxyoctadecadienoic acids (HODEs)	Exposure to DE led to systemic pro-oxidative effects characterized by increased lipid peroxidation and alteration of HDL protective capacities. DE effects on HDL antioxidant capacity were negatively correlated with PON activity, but positively correlated with levels of plasma 8-isoprostanes, 12-HETEs, 13-HODEs, liver MDA, and accompanied by perturbed HDL anti-inflammatory capacity and activation of the 5-lipoxygenase pathway in the liver. PON1 activity was significantly reduced among the DE-exposed mice compared to the FA group. No significant association was found with MPO.	DE exposure induced generation of dysfunctional pro-oxidative HDL, without affecting HDL-C levels. Several markers of lipid peroxidation in the blood and liver strongly correlated with the degree of HDL dysfunction.
**Feng et al. 2019, China** [34]	Male Wistar rats (6 weeks)	Rats were divided into 4 groups (8/group) by exercise status (sedentary vs. exercised) and PM_2.5_ exposure (instilled vs. non-instilled). All: *n* = 32	PM_2.5_	PM_2.5_ (3 mg/kg) on day 1, 3 and 5 in week 7. PM_2.5_ sample was collected in Beijing, China. Rats were exposed via intratracheal installation.	HDL-C level HDL cholesterol efflux capacity (CEC) HDL oxidization index (HDL-OI)	The levels of HDL-C, HDL-CEC and HDL-OI showed no significant changes between instilled vs. non-instilled rats, indicating that PM_2.5_ instillation did not significantly alter HDL function.	PM_2.5_ instillation showed limited adverse impact on HDL function (CEC, HOI), including HDL-C level.

*Studies are shown in chronological order based on the year of publication*

### Epidemiological studies

Results from a study by Mathew et al. showed that exposure to even low levels of PM_2.5_ (9,1 ± 1,8 μg/m^3^) for a short period of time were linked to an impaired HDL functionality measured as cholesterol efflux capacity (*n* = 50) [27]. In the Beijing AIRCHD study, participants were followed up with 4 study visits in a 14-month period (*n* = 73). Average daily concentration of ambient PM_2.5_ was high (62,9 μg/m^3^ (8,1–331,0 μg/m^3^)). Significant decreases in HDL cholesterol efflux capacity was associated with interquartile range increases in moving average concentrations of PM_2.5_ during the 1 to 7 days before each participant’s clinical visit. Higher ambient air pollutant levels were also associated with elevated HDL oxidation index (HOI) as well as reduced circulating levels of HDL-C. Furthermore, parameters of systemic oxidative stress and inflammation were found elevated in blood (ox-LDL, malondialdehyde and high-sensitivity C-reactive protein (hs-CRP)) [28], supporting the theory that this may be the mechanistic pathway for PM’s hazardous effects on HDL.

### Clinical trials

Maiseyeu et al. were the first to investigate the effects of PM on HDL function in humans (*n* = 32) [29]. Their controlled exposure study showed that brief inhalation of coarse PM (76,2 ± 51,5 μg/m^3^) from a rural source did not lead to development of HDL dysfunction as no alterations were found in HDL-cholesterol efflux capacity, HDL oxidation index or paraoxonase (PON) activity. The role of fine PM and impaired HDL functionality was studied by Ramanathan et al. in a clinical trial that explored the effects of fine PM (150 μg/m^3^) on HDL functionality in humans (*n* = 30) [30]. They found that brief exposures to concentrated PM_2.5_ induced acute adverse effects on the antioxidative properties of HDL (measured as HDL oxidation index). The changes in HDL functionality were, however, small, transient, and of short duration. A brief exposure to PM_2.5_ induced negative effects on HDL functionality that resolved within 24 h after exposure among those with lower HDL oxidation index pre-exposure values. They also assessed paraoxonase activity but did not find any alterations. The PM_2.5_ levels used in the study were considered significantly higher than in most cities but might be observed for short durations of time.

### Animal studies

Araujo et al. reported that the degree of HDL function seems to be dependent on particle-size as ultrafine (113 μg/m^3^) led to a greater degree of HDL dysfunction (measured as impaired anti-inflammatory capacity) than PM_2.5_ (439 μg/m^3^) in an experiment with Apo-E-deficient mice after whole-body exposure (for 40 days) [31]. Additionally, ultrafine particles resulted in larger early atherosclerotic lesions compared to PM_2.5_. The alterations in HDL functionality were found in the absence of changes in HDL-C levels. An experimental study by Li et al. showed that inhalation exposure to ultrafine particles (360 μg/m^3^ for 10 weeks) promoted pro-atherogenic lipid metabolism and reduced HDL antioxidative properties (significantly increased HDL oxidation index) in fat-fed LDL receptor-null mice [32]. The decreased antioxidative capacity of HDL was associated with a decrease in paraoxonase activity. Experiments measuring monocyte chemotaxis show marked correlation between impairment of HDL-antioxidant property and impaired anti-inflammatory feature [33, 35]. Hence, HDL oxidation index may serve as a measure of both HDL antioxidant and anti-inflammatory features [30]. A study by Yin et al., where Apo-E-deficient mice were exposed to diesel exhaust (DE) by simple inhalation (≈250 μg/m^3^ of PM_2.5_ for 2 weeks) revealed an induction of dysfunctional pro-oxidative HDL without affecting the quantitative levels [33]. Several markers of lipid peroxidation in the blood and liver were strongly correlated with the degree of dysfunction. Paraoxonase activity was found to be significantly reduced, whereas no significant association was found with myeloperoxidase. A recent study aimed to investigate the preventive effect of exercise training on vascular endothelial dysfunction induced by fine PM [34]. Exercise training promoted HDL function, whereas exposure to PM_2.5_ (3 mg/kg on day 1, 3 and 5) did not significantly alter HDL function in Wistar rats. It is, however, important to note that the route of exposure was intratracheal installation and not inhalation as in the previously featured studies.

## Discussion

**Fig. 2:**
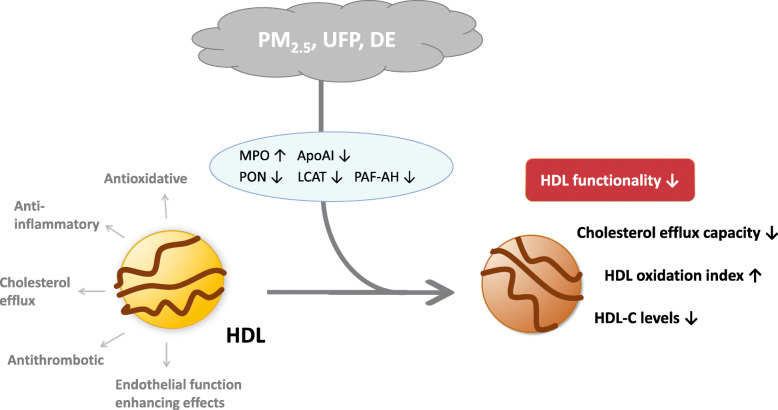
Suggested mechanistic pathway by which PM_2.5_, UFP and DE may change HDL-C levels and HDL functionality via effects on myeloperoxidase (MPO), apolipoprotein AI (ApoAI), paraoxonase (PON), lecithin cholesterol acyltransferase (LCAT) and platelet activating factor acetyl hydrolase (PAF-AH)

The current literature on PM-exposure and HDL functionality suggest that fine and ultrafine PM may impair functional properties of HDL via oxidative pathways [27, 28, 30–33]. Accumulating evidence links particles to atherosclerosis, in particular those with a high amount of organic chemicals [3, 6, 31, 36]. The particles may directly or indirectly react with protein and lipid components of HDL resulting in a dysfunctional phenotype without atheroprotective features (Fig. 2). In some studies, PM exposure was found to be associated with reduced HDL-C levels as well as altered HDL functionality [28, 32]. However, other studies report alteration in HDL functional properties without affecting the quantitative levels [31, 33].

### HDL functionality

A number of studies have found elevated PM exposure to be negatively correlated with the level of HDL-C [28, 32, 37–43]. However, the literature is somewhat inconsistent on this issue, as some studies find exposure to PM associated with an increase in HDL-C [44–46], while others find no statistical association [47–50]. Various studies have shown a significant increased incidence of atherosclerosis in individuals despite elevated levels of HDL-C [51]. Furthermore, HDL-C level raising therapies, as applied in multiple clinical trials, have failed to reduce CV events [52]. This may indicate that the HDL-C levels as such are not an obligatory link between PM and atherosclerosis.

Some have suggested that HDL particle number (HDL-P) may be a better indicator of the atheroprotective effect than HDL-C levels alone [53]. In a cross-sectional study, Bell et al. reported significant decreases in HDL particle number in relation to PM_2.5_ exposure [49]. They suggest that a reduction in smaller HDL particles supports the notion that changes in cholesterol efflux capacity might explain the link between air pollution and CVD, as the smaller particles accept cholesterol more efficiently than larger particles [54].

Other researchers have hypothesized that not all HDL is functionally similar. They suggest that the HDL functional properties might play a more significant role in atheroprotection than the cholesterol level itself [55]. HDL may render into a form characterized by impaired cholesterol efflux capacity. This dysfunctional form has reduced anti-inflammatory or even gained pro-inflammatory properties. Such changes have been seen in clinical conditions associated with inflammation and oxidative stress [51]. Accordingly, dysfunctional HDL has been found to be associated with increased incidence of CVD [51, 56]. For this reason, recent studies have placed focus on HDL function independent of cholesterol levels.

### Mechanistic considerations

A number of in vitro and in vivo studies have proven that pro-inflammatory and oxidative stimuli can rapidly convert HDL to a dysfunctional form, whereby it loses its atheroprotective features. The changes in HDL function are transient and more easily detected in individuals with high antioxidative capacity [30]. During acute-phase response, proteins like serum amyloid A (SAA) and ceruloplasmin bind to HDL which might limit its ability to promote cholesterol efflux as well as the antioxidant capacity [57]. Systemic inflammation can also impede functional properties of HDL by changing the proteome and lipidome [25]. HDL components, such as apolipoproteins and enzymes, might be targets for oxidative modifications that stimulate atherosclerotic processes [6, 58]. Thus, it is tempting to speculate that PM may directly or indirectly have similar effects. Myeloperoxidase is found to be elevated with increased PM exposure [59–61]. As myeloperoxidase induces oxidative damage on biological molecules, one plausible pathway for PM’s adverse effects on HDL function may be through myeloperoxidase-induced oxidative modification [27]. Oxidation of apolipoprotein-AI (Apo-AI) by myeloperoxidase and other reactive intermediates, weakens its capacity to promote reverse cholesterol transport. Oxidized Apo-AI is unable to activate lecithin cholesterol acyltransferase (LCAT) which is important for increasing the cholesterol efflux capacity [17, 62]. However, reduced HDL functionality without a corresponding increase in myeloperoxidase activity has been reported [27].

In addition to the apolipoproteins, HDL-associated enzymes like paraoxonase, lecithin cholesterol acyltransferase and platelet activating factor acetyl hydrolase (PAF-AH) contribute to the antioxidative features of HDL [23]. Paraoxonase is important in the protection of LDL from oxidative damage, and its expression seems to be downregulated in the presence of oxidative stress [63]. Studies in both animal and human find an association between decreased paraoxonase activity and increased risk of developing atherosclerosis [64]. Accordingly, in LDLR−/− mice exposed to ultrafine particles, a decreased paraoxonase activity was associated with a larger atherosclerotic lesion size [32, 33]. However, other studies did not observe altered paraoxonase activity following exposure to PM [29, 30].

Exposure to PM has been associated with development of dysfunctional HDL in several studies [27, 28, 30–33]. Association was found for ultrafine and fine PM, and the effects of PM on HDL functionality seem to be associated with smaller particle size. This is further supported by the finding in one small study where coarse PM was not associated with impaired HDL functionality [29]. Ultrafine particles also seem to give larger early atherosclerotic lesions in mice than PM_2.5_. This may in part be due to a larger deposition in the lower airways, but the relative smaller size of ultrafine particles will also result in higher particle number and larger surface per mass [31, 65, 66]. Irrespectively, it is interesting to note that ultrafine particles not only produced more proatherogenic effects than PM_2.5_, but also resulted in a larger degree of HDL dysfunction. Thus, this finding supports the hypothesis of HDL dysfunction as a mechanistic link between PM exposure and atherosclerosis.

In addition to studies assessing the hazardous effects of PM on HDL, there is also a study evaluating the protective capacity of HDL against exposure to PM [67]. The results showed that unmodified HDL inhibited oxidative effects in bovine aortic endothelial cells and RAW264.7 macrophages exposed to diesel exhaust particles in vitro. In contrast, dysfunctional HDL failed to inhibit diesel exhaust particle-induced oxidation and oxidative cellular effects and instead, the exposure promoted further oxidation. These results strengthen the hypothesis that normal HDL protect against adverse effects of air pollution.

### Susceptibility and co-exposures

There are genetically susceptible individuals with low antioxidative capacity in the blood which might represent a population with an increased risk of PM-related CVD. Some genetic disorders which alter critical enzymes, lipid transfer proteins or receptors crucial for the metabolism and function of HDL may impair the functional aspects of the lipoprotein [68]. The well-established risk factors for atherosclerosis - hypertension, dyslipidaemia and obesity – are all characterized by systemic inflammation and oxidative stress [69]. Such conditions might explain why HDL renders dysfunctional in patients with metabolic syndrome [70].

It is interesting to note that cigarette smoking has also been a suggested contributor to CVD via alterations in lipid profiles with impact on HDL [71]. Evidence suggests that smoking reduces HDL-C levels by altering critical enzymes of lipid transport. Smoking is reported to reduce the activity of lecithin cholesterol acyltransferase, which might impede maturation of HDL and lead to a rapid clearance of nascent HDL [56]. The anti-oxidative and anti-inflammatory capacity of HDL, as well as the cholesterol efflux capacity, have been found to be impaired by cigarette smoking [72]. It is well documented that smoking cessation reduces the risk of CVD. Interestingly, smoking cessation improves HDL functionality in coronary artery disease patients in the absence of changes in HDL-C levels, Apo-AI levels or HDL subfractions [72, 73]. Overall, smoking-induced reduction in HDL-C levels and in particular, HDL function seems to be important for atherosclerosis. It is tempting to hypothesize that a similar mechanism may also apply to PM in ambient air and that smokers may represent a particularly sensitive group.

### Methodological considerations

#### Human exposure and epidemiological studies

Particulate matter air pollution as a risk factor for CVD is relatively modest compared to other well-established factors linked to personal lifestyle, including physical inactivity, unhealthy diet, smoking and excessive alcohol use [1]. Nonetheless, the ubiquitous nature and therefore the magnitude of populations affected, makes PM air pollution a serious threat to human health. Overall, the epidemiological and clinical data suggest a possible association between PM-exposure and dysfunctional HDL. The number of available studies is limited and focus only on the effects of short-term particle exposure. A major strength of the studies is, however, their strong research designs as randomized controlled trials and prospective follow-up studies. The inclusion of non-smoking populations limits confounding by tobacco smoke.

#### Animal studies

There are obvious genetic and environmental factors to take into consideration when extrapolating from animal studies to human. Most importantly, atherosclerosis is not as common in rodents as in humans. Even in the often-used atherosclerosis-prone ApoE−/− (knock-down) mouse model, plaques formed do not rupture [74]. Regarding air pollution particles, the horizontally-positioned respiratory system in rodents presents obvious problematic implications for particle deposition and removal. Thus, results from animal experiments are not directly comparable with the health effects observed in human studies. However, the impact of animal studies may be improved with the combined use of human in vitro studies on specific processes in cells considered to be central in atherosclerosis.

## Conclusion

Epidemiological and clinical studies when combined with experimental animal and in vitro studies, support the notion that fine and ultrafine PM may promote HDL dysfunction via oxidative pathways. Combined, these studies suggest a causal pathway between air pollution, PM-induced dysfunctional HDL and atherosclerosis. Several mechanisms have been proposed, but the underlying biological pathways remain to be fully elucidated. One central theory suggests PM promotes atherosclerosis as a result of its systemic oxidative and inflammatory effects. This type of inflammatory milieu can affect plasma lipoproteins and may increase the atherogenic effect of LDL while reducing the atheroprotective effect of HDL. Recent studies have challenged the well-established idea that higher levels of HDL-C are always beneficial and lower levels of HDL-C are always detrimental. Ultimately, it has become clear that the functional properties of HDL are more important than the levels per se [75]. As shown in this review, there are studies suggesting that fine and ultrafine PM may promote HDL dysfunction via oxidative pathways. By affecting the protein and lipid components of HDL, cholesterol efflux capacity, as well as other anti-oxidative and anti-inflammatory features might be impaired.

### Perspectives and future studies

As the current literature on PM exposure and HDL function is very limited, there is a need for more epidemiological as well as experimental studies to strengthen any possible causal relationship and determine any association to atherosclerosis as well as its possible underlying mechanisms. It would be interesting to explore whether short-time exposures can induce changes in HDL function or whether long-term exposures are essential. Studies so far have only been assessing the role of PM on HDL function in healthy populations. It is thus important to study genetically susceptible groups or other groups considered to have an increased risk of PM-related CVD like the elderly, individuals with metabolic syndrome, and those with diabetes [1]. Assessing HDL function in individuals with high exposure from ambient air or due to occupational settings (e.g. truck drivers or welders) might give additional insight. On the clinical side, one should examine how HDL functionality in CVD-patients is affected by PM exposure, and explore the efficacy of preventive measures like treatment with statins and antioxidants. Such studies may yield a better understanding of the PM-mediated pathogenesis and may lead to the identification of new biomarkers of PM-induced systemic effects, as well as a potential therapeutic target for treatment of atherosclerosis.

## Data Availability

Not applicable.

## Abbreviations

ABCA1: ATP-binding cassette transporter A1
ABCG1: ATP-binding cassette transporter G1
Apo: Apolipoprotein
CEC: Cholesterol efflux capacity
CETP: Cholesterol ester transfer protein
CV: Cardiovascular
CVD: Cardiovascular disease
DE: Diesel exhaust
FA: Filtered air
HDL: High-density lipoprotein
HDL-C: High-density lipoprotein cholesterol
HOI: High-density lipoprotein oxidation index
hs-CRP: High-sensitivity C-reactive protein
IL-1β: Interleukin-1β
IL-6: Interleukin-6
LCAT: Lecithin-cholesterol acyltransferase
LDL: Low-density lipoprotein
LDL-C: Low-density lipoprotein cholesterol
MPO: Myeloperoxidase
ox-HDL: Oxidized high-density lipoprotein
ox-LDL: Oxidized low-density lipoprotein
PAF-AH: Platelet activating factor acetyl hydrolase
PAH: Polycyclic aromatic hydrocarbons
PM: Particulate matter
PON: Paraoxonase
RCT: Reverse cholesterol transport
ROS: Reactive oxygen species
SAA: Serum amyloid A
TNF-α: Tumor necrosis factor-α
UFP: Ultrafine particles
VLDL: Very-low-density lipoprotein
